# Enhancer-LSTMAtt: A Bi-LSTM and Attention-Based Deep Learning Method for Enhancer Recognition

**DOI:** 10.3390/biom12070995

**Published:** 2022-07-17

**Authors:** Guohua Huang, Wei Luo, Guiyang Zhang, Peijie Zheng, Yuhua Yao, Jianyi Lyu, Yuewu Liu, Dong-Qing Wei

**Affiliations:** 1 School of Electrical Engineering, Shaoyang University, Shaoyang 422000, China; qq25822@163.com (W.L.); guiyang9542@163.com (G.Z.); zhengpeijie1997@163.com (P.Z.); ljy990309@163.com (J.L.); 2 School of Mathematics and Statistics, Hainan Normal University, Haikou 571158, China; yaoyuhua2288@163.com; 3 College of Information and Intelligence, Hunan Agricultural University, Changsha 410083, China; yuewuliu@whu.edu.cn; 4 State Key Laboratory of Microbial Metabolism, and School of Life Sciences and Biotechnology, Shanghai Jiao Tong University, Shanghai 200240, China; dqwei@sjtu.edu.cn

**Keywords:** enhancer, promoter, deep learning, feed-forward attention, convolution neural network, long-short term memory, residual neural network

## Abstract

Enhancers are short DNA segments that play a key role in biological processes, such as accelerating transcription of target genes. Since the enhancer resides anywhere in a genome sequence, it is difficult to precisely identify enhancers. We presented a bi-directional long-short term memory (Bi-LSTM) and attention-based deep learning method (Enhancer-LSTMAtt) for enhancer recognition. Enhancer-LSTMAtt is an end-to-end deep learning model that consists mainly of deep residual neural network, Bi-LSTM, and feed-forward attention. We extensively compared the Enhancer-LSTMAtt with 19 state-of-the-art methods by 5-fold cross validation, 10-fold cross validation and independent test. Enhancer-LSTMAtt achieved competitive performances, especially in the independent test. We realized Enhancer-LSTMAtt into a user-friendly web application. Enhancer-LSTMAtt is applicable not only to recognizing enhancers, but also to distinguishing strong enhancer from weak enhancers. Enhancer-LSTMAtt is believed to become a promising tool for identifying enhancers.

## 1. Introduction

Enhancers are short pieces of DNA sequences of 50 to 1500 bp, which can accelerate the transcription of target genes by binding the transcription factors [1,2]. Unlike the promoters, enhancers are located either in the upstream/downstream or within the genes they regulate and doesn’t have to be close to the starting sites of transcription [2,3,4]. Increasing evidences indicate that enhancers play a critical role in the gene regulation [4,5]. The enhancers control the expression of genes involved in cell differentiation [6,7] and are responsible for morphological changes in three spine stickleback fish [8]. The enhancers orchestrate critical cellular events such as differentiation [9,10], maintenance of cell identity [11,12], and response to stimuli [13,14,15] by binding to transcription factors [16]. The enhancers are closely related to inflammation and cancer [17]. Therefore, precisely detecting enhancers from DNA sequences is critical to further investigate their functions or roles in the cellular processes.

The methods or techniques used to identify enhancers are divided into two categories: high-throughput experimental technology and computational method [5,18]. The former includes chromatin immunoprecipitation followed by deep sequencing (ChIP–seq) [19,20], protein-binding microarrays (PBMs) [21], systematic evolution of ligands by exponential enrichment (SELEX) [22], yeast-one-hybrid (Y1H) [23], and bacterial-one-hybrid [24]. The main idea behind these technologies is to identify enhancers by recognizing properties of the enhancer-binding interactors [16]. There are generally four ways of experimental technology. The first way is to identify enhancers by binding sites of the specific transcription factors (TFs) with the help of ChIP-seq [13,25]. These techniques are restricted to cell-type or tissue-specific TFs. The second way is to detect enhancers by recognizing the binding sites of transcriptional co-activators such as CBP (also known as CREB-binding protein or CREBBP) and P300 (also called EP300 or E1A binding protein p300) recruited by the TFs [12,13,26]. However, not all enhancers are characterized by the co-activators, and the ChIP-grade antibodies are not always available. The third way is to identify nucleosome-depleted open regions of DNase I hypersensitivity [27]. The open regions include other DNA elements, such as promoters, silencers/repressors, insulators, and other function-unknown sequences [28,29]. The modifications of histones in the flanking nucleosomes are of certain signature of enhancers. For example, histones flanking active enhancers are typically marked by H3 mono-methylated at lysine 4 (H3K4me1), while histone flanking active promoters are marked by H3K4me3 [13]. Therefore, the fourth method is genome-wide mapping of histone modifications. In spite of great success in identifying enhancers, high-throughput experimental technologies have two drawbacks: they are time-consuming and expensive. Therefore, it is a challenging task to identify all enhancers from thousands of tissues or cells.

The computational methods have been developed to complement the high-throughput experimental technologies over the recent decade [18,30,31]. The computational methods include genomics comparison-based methods and machine learning-based methods. The enhancers reside in any region of the genome, so it is very difficult to find intuitively linear motifs of enhancers by genomics comparison-based methods. Machine learning-based methods build a classification model to fit known enhancers and then predict new enhancers. Furthermore, Machine learning-based methods are capable of discovering non-linear hidden motifs of enhancers. To date, there are at least twenty machine learning-based methods for enhancer prediction [16,32,33,34,35,36,37,38,39,40,41,42,43,44,45,46,47,48,49,50,51,52,53,54,55,56,57,58,59,60,61,62,63], such as iEnhancer-2L [40], iEnhancer-PsedeKNC [41], EnhancerPred [42], and EnhancerPred2.0 [43]. The general workflow of these methods is firstly to compute representation of sequences such as pseudo k-tuple nucleotide composition, nucleotide binary profiles, as well as accumulated nucleotide frequency, then to learn a classifier by using a machine learning algorithm such as support vector machine and random forest, and finally to predict unknown sequences.

The aforementioned machine learning-based methods require sophisticated design of representations as well as sophisticated selection of conventional machine learning algorithms. In practice, any single representation is not able to characterize enhancers well, while a combination of diverse representations has the potential to improve the performance but reduces the generalization ability of the methods. The deep learning methods that have been developed in the recent decades have proven to be good at addressing complex issues, including protein structure prediction, which is thought to be one of the most challenging tasks [64,65]. Yao et al. [60] presented a word embedding-based deep learning method named iEnhancer-GAN to detect enhancers. To make up for insufficiency of the number of training samples, iEnhancer-GAN [60] used the sequence generative adversarial net [66] to augment training samples. Min et al. [33] developed a deep convolution neural network (CNN)-based method for distinguishing enhancers from non-enhancers, which required only primary sequences as input. Khanal et al. [52] exploited word embedding in the field of natural language processing as well as CNN to construct a method named iEnhancer-CNN. Nguyen et al. [50] integrated multiple CNNs into the iEnhancer-ECNN. The CNN is capable of characterizing local properties [67], but is insufficient to represent semantic relationships between words in the context of sequences. Tan et al. [48] exploited recurrent neural networks (RNN) and integrated the output of both RNN and CNN for the final decision. Le et al. [55] presented an advanced method (BERT [68]) to capture semantics of DNA sequences. On the basis of analysis of the published works or methods for detecting enhancers, we presented a bi-directional long-short term memory (Bi-LSTM) and attention-based deep learning method for enhancer recognition called Enhancer-LSTMAtt.

## 2. Data

For fair comparison with the state-of-the-art methods, we used the same benchmark dataset as those in iEnhancer-2L [40], iEnhancer-PsedeKNC [41], EnhancerPred [42], EnhancerPred2.0 [43], Enhancer-Tri-N [44], iEnhaner-2L-Hybrid [45], iEnhancer-EL [46], iEnhancer-5Step [47], DeployEnhancer [48], ES-ARCNN [49], iEnhancer-ECNN [50], EnhancerP-2L [51], iEnhancer-CNN [52], iEnhancer-XG [53], Enhancer-DRRNN [54], Enhancer-BERT [55], iEnhancer-KL [56], iEnhancer-RF [57], spEnhancer [58], iEnhancer-EBLSTM [59], iEnhancer-GAN [60], piEnPred [61], iEnhancer-RD [62], and iEnhancer-MFGBDT [63]. The dataset was initially collected by Liu et al. [40] from chromatin state information of nine cell lines (H1ES, K562,GM12878, HepG2, HUVEC, HSMM, NHLF, NHEK and HME) which was annotated by ChromHMM [69,70]. The initial enhancers included sequences of less than 200 bp and were of high homologies. In order to comply with the length of nucleosome and linker DNA, less than 200 bp sequences were removed and more than 200 bp sequences were cut into segments of fixed length (200 bp). Liu et al. [40] employed the CD-HIT to decrease or remove homologies among sequences. The CD-HIT [71,72,73] is a clustering tool to reduce redundant sequences. The generated non-redundant sequences were used to examine the dependency of methods on homology [74,75,76,77,78,79,80]. Liu et al. [40] set sequence identity to 0.8, indicating that homologies between chosen enhancers were not less than 0.8. The enhancers were grouped into strong enhancers and weak enhancers. The numbers of weak enhancers and the non-enhancers are much greater than those of strong enhancers. To achieve a balance between positive and negative samples, Liu et al. [40] randomly sampled the same numbers of weak enhancers as the strong enhancers and the same number of non-enhancers as the sum of strong and weak enhancers. The benchmark dataset S consisted of the strong enhancer set $S_{\mathrm{strong}}$, the weak enhancer set $S_{\mathrm{weak}}$, and the non-enhancer set $S_{\mathrm{non}}$, whose numbers were 742, 742, and 1484, respectively. During the process of distinguishing the enhancer from the non-enhancer, both the strong enhancers and the weak enhancers were viewed as positive samples, and the non-enhancers were viewed as negative samples. During the process of distinguishing strong enhancers from weak enhancers, strong enhancers were positive, and weak enhancers were negative.

Another dataset $S^{i}$ was used for the independent test, which was from reference [46]. The $S^{i}$ contained 100 strong enhancers $S_{\mathrm{strong}}^{i}$, 100 weak enhancers $S_{\mathrm{weak}}^{i}$ and 100 non-enhancers $S_{\mathrm{non}}^{i}$. The sequence identities between any two enhancers are not more than 0.8 by processing by CD-HIT [71,72,73].

## 3. Methods

**Figure 1:**
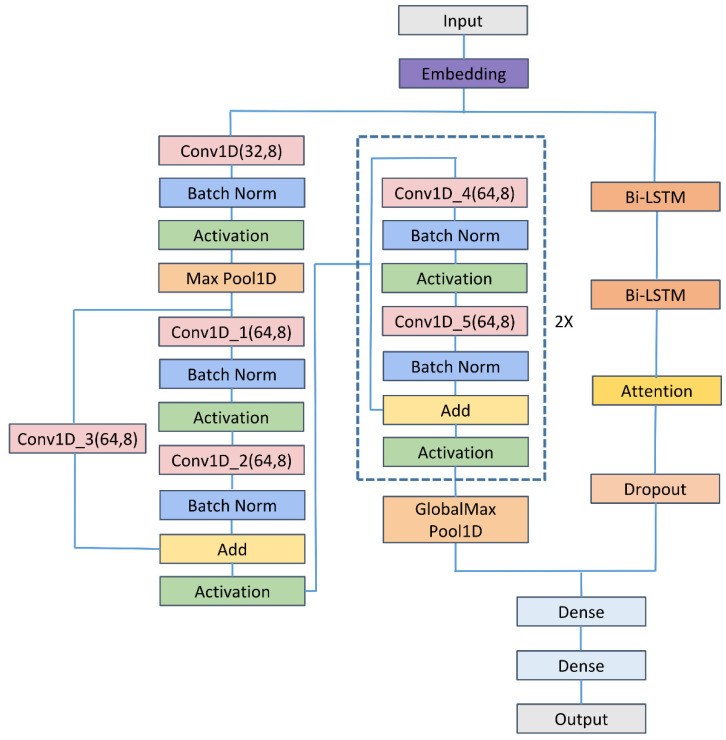
The architecture of the Enhancer-LSTMAtt. Conv1D, Batch Norm, Attention, Activation, Dense, and Max Pool 1D denote the 1D CNN layer, the batch normalization layer, the feed-forward attention layer, activation function, the fully-connected layer, and the max pooing layer respectively.

As shown in Figure 1, the proposed method comprised mainly input, embedding, 1D CNN, residual neural network (ResNet), Bi-LSTM, attention, dropout, flattened, and fully connected layers. The input was DNA segments of 200 bp. Then, DNA segments were transformed into number sequences by

(1) $$f\left(X\right)=\{\begin{array}{cc} \begin{array}{c} 0\\ 1\\ \begin{array}{c} 2\\ \begin{array}{c} 3\\ 4 \end{array} \end{array} \end{array} & \begin{array}{c} A\\ C\\ \begin{array}{c} G\\ \begin{array}{c} T\\ N \end{array} \end{array} \end{array} \end{array}$$

where N denoted the characters of the unknown nucleotide. The embedding of number sequences was entered into the convolution module and the LSTM module. The convolution module consisted mainly of the 1D CNN and ResNet [81,82,83], while the LSTM module comprised mainly Bi-LSTM [84,85] and feed-forward attention [86,87]. The concatenation of outputs of the two modules was entered into the fully connected layer. Following the fully connected layer was the final layer, which contained one neuron representing the probabilities of belonging to enhancers. We set the threshold to 0.5, and thus more than 0.5 output indicated that the corresponding input was predicted to be positive and otherwise to be negative. The numbers of the parameters and the shape of output in each layer of the Enhancer-LSTMAtt were listed in Table 1.

### 3.1. Embedding Layer

The embedding is generally the first layer of the deep neural network, whose role is to map the categorical (discrete) variable to continuous vectors (https://towardsdatascience.com/neural-network-embeddings-explained-4d028e6f0526 (accessed on 3 March 2022)) [88]. The traditional one-hot encoding suffered from two defaults. One was that it was not capable of distinguishing similarities between representations. Another was that the representation was sparse in the case of the large vocabulary. The embedding well solved two issues and thus was widely applied to the area of natural language processing. The embedding can be used alone, such as word2vec and Glove, or fused into the deep neural network as the first layer.

### 3.2. CNN

**Figure 2:**
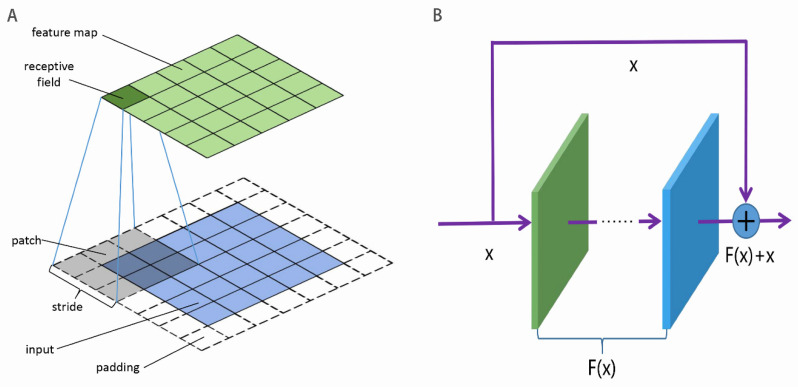
Illustration of (**A**) convolutions in the CNN and (**B**) the ResNet unit. F(x) generally is CNN.

CNN is one of most popular neural network architectures used to construct deep neural network [67,89,90]. The main characteristic of the CNN is to capture the local hidden structure by using convolutional kernels or filters. As shown in Figure 2A, the input is divided into patches, which are convoluted into the feature map by the convolutional kernel. The patches are allowed to overlap, and the interval between adjacent patches is called the stride. All of the patches in the same input share the convolutional kernel which are learnable parameters. To keep the size of the input unchanged, the input is sometimes required to pad. To increase the non-linear ability of the CNN, the activation function is added to the feature map. The activation function includes ReLU, sigmoid, tanh, weakly ReLU, and ELU. The pooling in the CNN is a non-linear down-sampling, whose role is to reduce the dimensionality of representations and to speed up the calculation. In addition, the pooling is able to avoid or decrease the over-fitting issue.

### 3.3. ResNet

As the number of stacked layers in the deep neural network increased, three issues would occur: information loss, gradient vanishing or exploding, and network degradation. This resulted in the worse performance of the deep neural network [89]. He el al. [81] presented ResNet to address these issues. The basic architecture of ResNet [81] was composed of residual mapping F(x) and identity mapping x, as shown in Figure 2B. The identify mapping ensured no loss of inputted information in spite of increasing layers. The residual mapping was viewed as the learnable residual function and might be conventional convolutions. The ResNet enabled the neural network to go deeper without network degradation. Li et al. [81] used ResNet to construct a 152-layers deep network, which reduced the top 5 error rates of image recognition to 5.71% on the ImageNet.

### 3.4. Bi-LSTM

**Figure 3:**
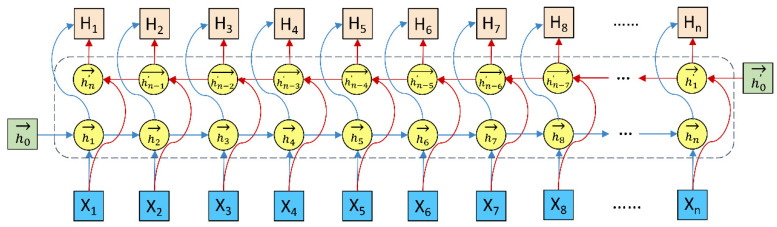
The diagram of the Bi-LSTM unit. $X_{i}$, $\vec{h_{i}}$, $\vec{h_{n-i}^{\prime }}$, and $H_{i}$ denoted the input, the forward hidden state, the backward hidden state, and the bi-directional hidden state at the time step i, respectively.

Long-short term memory (LSTM) [90] is a type of recurrent neural network (RNN) [91,92]. The RNN is especially suitable to deal with time series questions due to its architecture: sharing weights at all of the time steps. The RNN was applied to a wide range of fields, including speech recognition [93], continuous B-cell epitope prediction [94], sentiment analysis [95], and action recognition [96]. The major default of the RNN was that it is prone to cause gradient vanishing or exploding for long sequence analysis. Therefore, the RNN was restricted to short sequences [97,98]. The LSTM [90] employed the gate mechanism to control conveying of information, including selective addition of new information or removal of information accumulated previously. The LSTM was able to capture the relationship of the words in the former with those in the back but was not able to characterize the relationship of the words in the back with those in the former. The Bi-LSTM [84,85] addressed the issue well. As shown in Figure 3, the Bi-LSTM was made up of two LSTMs, one from forward to backward and another from backward to forward. The two LSTMs shared embedding of words but were independent of each other in terms of learnable parameters. The concatenation of hidden states in both LSTMs corresponded to the output of the Bi-LSTM.

### 3.5. Feed-Forward Attention

Attention mechanisms are increasingly becoming a hot topic in the field of deep learning. The attention mechanisms are a scheme of allocating weights, which is very similar to the scene where one assigns a different focus to different parts when watching an object. There are many attention schemes, including feed-forward attention [99] and self-attention [100], etc. The feed-forward attention is intended to make up for the deficiency of the LSTM in the long-term dependency. Assume that the hidden state at time step t in the LSTM was $h_{t}$. The context vector generated by the feed-forward attention was computed by

(2) $$c=\sum_{t=1}^{T}\alpha_{t}h_{t},$$

where $\alpha_{t}$ was the attention weight of the hidden state $h_{t}$. $\alpha_{t}$ was defined by

(3) $$\alpha_{t}=\frac{\exp\left(e_{t}\right)}{\sum_{k=1}^{T}\exp\left(e_{k}\right)},$$

where

(4) $$e_{t}=\delta \left(h_{t}\right).$$

δ was the learnable parameter.

### 3.6. Dropout Layer

Dropout proposed by Hinton et al. [101] is a concept to train deep neural network. In the process of training, a certain proportion of neurons are randomly dropped out, and all of the neurons are used as usual in the process of prediction [102]. The dropout serves two-fold functions: speeding up training of the deep neural network and reducing over-fitting.

### 3.7. Flatten Layer and Fully Connected Layer

The flatten layer was intended to convert the shape of data so as to link conveniently the next layers. The flatten layers did not have any learnable parameters. The fully connected layer was identical to the hidden layer in the multilayer perceptron, and each neuron was connected to all of the neurons in the previous layer. 

## 4. Cross Validation and Evaluation Metrics

To examine the predictive performance of the presented method, we used n-fold cross validation and independent test. In the n-fold cross-validation, the training dataset was divided into n parts of equal or approximately equal size, of which n-1 parts were used to train the model and the remaining part was used to test the model. This process was repeated n times. In the independent test, the training dataset was used to train the model, and the independent dataset was used to test the model.

This is a binary classification issue, so we used common metrics to evaluate the predictive performance, including sensitivity (SN), specificity (SP), accuracy (ACC), and Matthews’ correlation coefficient (MCC), which are defined as

(5) $$\mathrm{SN}=\frac{\mathrm{TP}}{\mathrm{TP}+\mathrm{FN}}$$

(6) $$\mathrm{SP}=\frac{\mathrm{TN}}{\mathrm{FP}+\mathrm{TN}}$$

(7) $$\mathrm{ACC}=\frac{\mathrm{TP}+\mathrm{TN}}{\mathrm{TP}+\mathrm{FN}+\mathrm{FP}+\mathrm{TN}}$$

(8) $$\mathrm{MCC}=\frac{\mathrm{TP}\times \mathrm{TN}-\mathrm{FP}\times \mathrm{FN}}{\sqrt{\left(\mathrm{TP}+\mathrm{FN}\right)\left(\mathrm{TP}+\mathrm{FP}\right)\left(\mathrm{TN}+\mathrm{FN}\right)\left(\mathrm{TN}+\mathrm{FP}\right)}}$$

where TP is the number of true positive samples, FN is the number of false negative samples, FP is the number of false positive samples, and TN is the number of true negative samples. SN, SP and ACC lie between 0 and 1. The MCC ranges from −1 to 1. More values of SN, SP, ACC and MCC indicated better performance.

The receiver operating characteristic (ROC) curve is a commonly used way to evaluate the performance of binary classification methods. The ROC curve is drawn by plotting the true positive rate (TPR) against the false positive rate (FPR) under various thresholds. The TPR and the FPR are computed by

(9) $$\mathrm{TPR}=\frac{\mathrm{TP}}{\mathrm{TP}+\mathrm{FN}}$$

(10) $$\mathrm{FPR}=\frac{\mathrm{FP}}{\mathrm{FP}+\mathrm{TN}}$$

The area under the ROC curve (AUC) ranges from 0 to 1. If the AUC was equal to 1, the prediction was perfect. The AUC equaling 0.5 indicated a random prediction and the AUC equaling to 0 was an opposite prediction.

We used Python programming language along with the deep learning toolkit TensorFlow (version 2.0) to implement the Enhancer-LSTMAtt. We conducted 5-fold cross validation, 10-fold cross validation and independent test on the Microsoft Windows 10 operating system, which is installed on a notebook computer with 32G RAM and 6 CPUs, each with 2.60 GHz. Each epoch costs about 25 s in the training process, while prediction of each sample takes no more than 2 s by using the trained Enhancer-LSTMAtt. The codes along with the datasets are available at Github: https://github.com/feng-123/Enhancer-LSTMAtt.

## 5. Results

**Figure 4:**
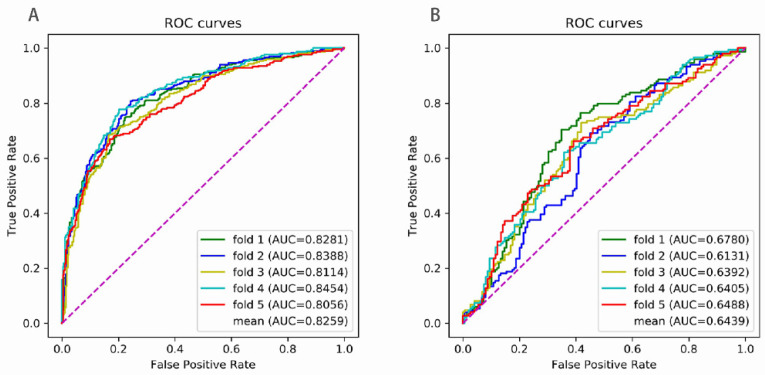
The ROC curves of 5-fold cross validation (**A**) for recognizing the enhancers and (**B**) for distinguishing strong from weak enhancers. The purple line is the baseline of ROC curve, named random guessing line.

We tested the Enhancer-LSTMAtt for its ability to not only distinguish between enhancers and non-enhancers, but also discriminate strong enhancers from weak enhancers. The process of distinguishing between enhancers and non-enhancers was called the first stage, where all of the enhancers, including weak enhancers, were positive samples. The process of discriminating strong from weak enhancers was called the second stage, where the strong enhancers were positive and the weak enhancers were negative samples. We conducted 5-fold cross validation in dataset S. Figure 4 shows the ROC curve of each fold, and Table 2 lists the evaluation of performance. We obtained an average AUC of 0.8259 in the first stage and an average AUC of 0.6439 in the second stage. We achieved an average SN of 0.7304, an average SP of 0.8006, an average ACC of 0.7655, and an average MCC of 0.5339 in the first stage and an average SN of 0.6765, an average SP of 0.6024, an average ACC of 0.6395, and an average MCC of 0.2804 in the second stage. Obviously, the predictive performance in the first stage was much better than that in the second stage, indicating that it was more difficult to discriminate strong enhancers from weak enhancers than to discriminate enhancers from non-enhancers.

### 5.1. Comparison with State-of-the-Art Methods

As mentioned in the introduction, no less than 20 computational methods have been developed for predicting enhancers. Some methods were tested by jackknife test, some by 5-fold cross validation, some by 10-fold cross-validation, and some by the independent test. Some methods distinguished enhancers from non-enhancers, while some discriminated strong from weak enhancers. Table 3 summarizes these methods. Since the jackknife test is too time-consuming for deep learning methods, we conducted 5-fold cross, 10-fold cross validation, and independent tests to compare these state-of-the-art methods. Table 4 and Table 5 list the evaluation of performances. Different indices evaluate different performances. For instance, SN is used to evaluate the ratio of the number of correctly predicted positive samples to the total number of positive ones, while SP is the ratio of the number of correctly predicted negative samples to the total number of negative ones. Sometimes, the two indices would not maintain synchronization, which was difficult to determine as good or bad. In this case, the overall indices could be used, such as ACC and MCC. In the 5-fold cross-validation, Enhancer-LSTMAtt was superior to Enhancer-BERT [55], DeployEnhancer [48] and iEnhancer-RF [57] in terms of ACC and MCC in the first stage and exceeded iEnhancer-PsedeKNC [41], DeployEnhancer [48], EnhancerP-2L [51], and iEnhancer-RF [57] in terms of MCC in the second stage. In the 10-fold cross-validation, Enhancer-LSTMAtt reached competitive performance with ES-ARCNN [49], iEnhancer-XG [53], and iEnhancer-MFGBDT [63] in the second stage.

**Figure 5:**
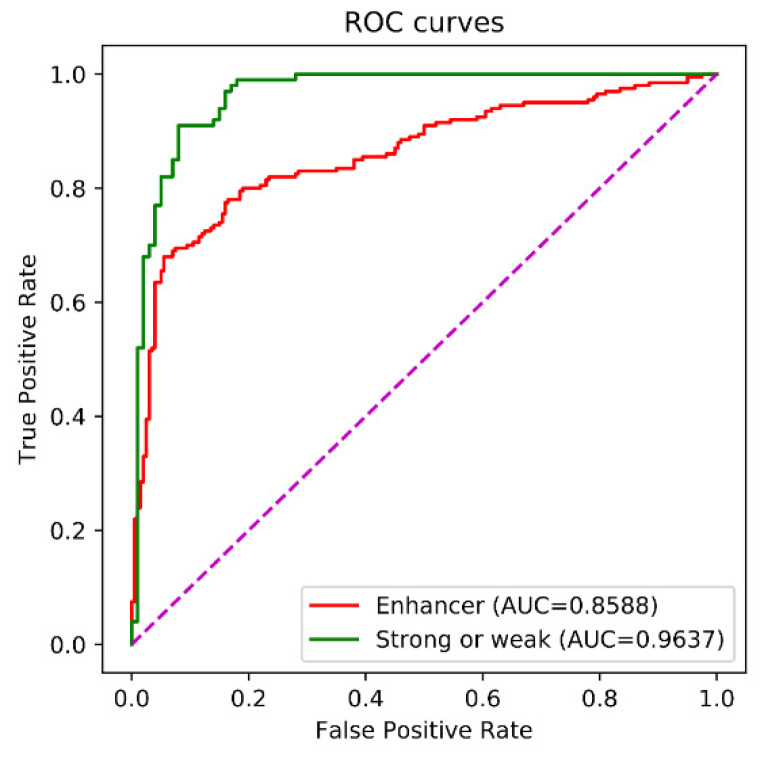
The ROC curves of the independent test. The purple line is the baseline of ROC curve, named random guess line.

Table 6 lists evaluation of performances of all of the 19 methods on the independent test. To the best of our knowledge, nearly all of the methods used the same independent dataset $S^{i}$ for independent test, and no other published enhancers were collected as the second independent dataset. Obviously, Enhancer-LSTMAtt achieved competitive performance with these state-of-the-art methods. In the first stage, Enhancer-LSTMAtt reached the best SP (0.8150), the best ACC (0.8050), and the best MCC (0.6101), achieved a second AUC (0.8588), which was less than the AUC of iEnhancer-RF, and obtained a competitive SN (0.7950), which was less than the SN of iEnhancer-GAN [60], spEnhancer [58], iEnhancer-5Step [47], piEnPred [61], iEnhancer-RD [62], and iEnhancer-BERT [55]. In the second stage, the Enhancer-LSTMAtt reached the best SN, ACC and MCC, a second AUC to that of the iEnhancer-RF [57], and a second SP to that of the Enhancer-DRRNN [54]. These results indicated that Enhancer-LSTMAtt is a competitive method to recognize enhancers. It must be pointed out that we didn’t conduct cross validation and independent test for 19 methods, and the evaluation of their performances directly came from their published papers. Figure 5 shows the ROC curve of the independent test.

### 5.2. Enhancer-LSTMAtt Webserver

We implemented Enhancer-LSTMAtt into a user-friendly web application which is freely available at http://www.biolscience.cn/Enhancer-LSTMAtt/ (accessed on 20 May 2022) to all of the scientific researchers. The web application is easy for users to use. The only thing users do is to upload DNA sequences in a FASTA format either by pasting it into textbox or by uploading a file. Users click the “submit” button, and then the web application returns prediction in the 7-tuple. The first column is the names of input sequences, the second is the range of the enhancers, the third and the fourth are the probabilities of predicting as the enhancer and the non-enhancer respectively, the fifth and the sixth are the probabilities of predicting as the strong and the weak enhancer, and the seventh is the predicted result.

### 5.3. Discussion

**Figure 6:**
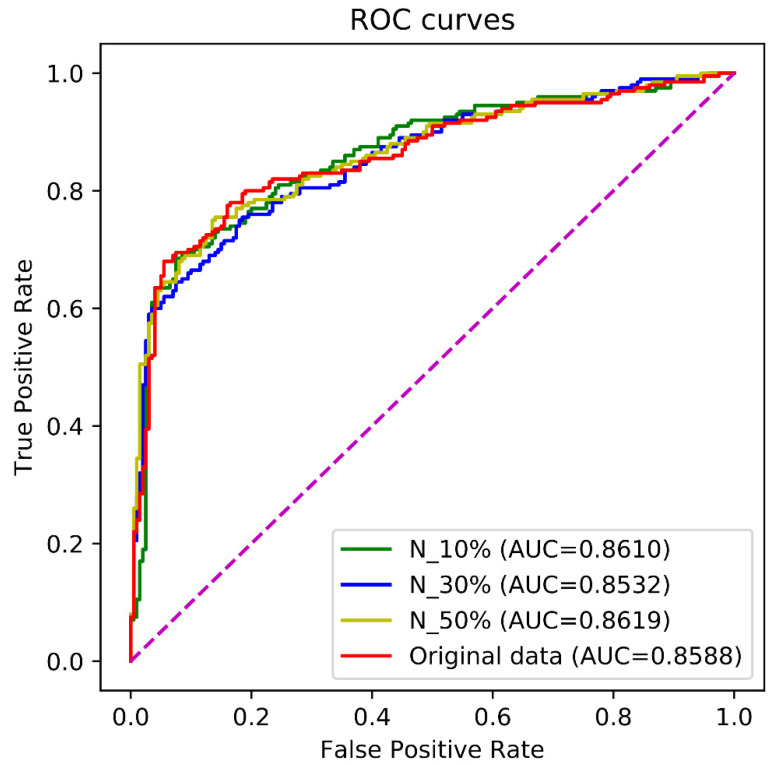
The ROC curves by the independent test over different non-enhancer sets. N_10, N_30, and N_50 denote the training sets in which 10%, 30%, 50% of non-enhancers were formed by mutation from the original non-enhancers, respectively. The original data denotes the training set which was made up of the enhancers and original non-enhancers. The purple line is the baseline of ROC curve, named random guessing line.

We investigated effect of different non-enhancers on the methods. Due to non-enhancers that were not available before sampling, we used the sampling and mutation strategy to generate new non-enhancers. We randomly selected 30%, 40%, and 50% of samples in the non-enhancer set $S_{\mathrm{non}}$ and made them mutate. The mutated non-enhancers and the non-mutated non-enhancers constituted three new non-enhancer sets which along with the enhancers further comprised three new training sets, respectively. We used the independent test to examine the performance of the proposed method trained by the new training sets. As shown in Figure 6, the non-enhancers have a certain influence on the performance of the method, but this influences is little.

**Figure 7:**
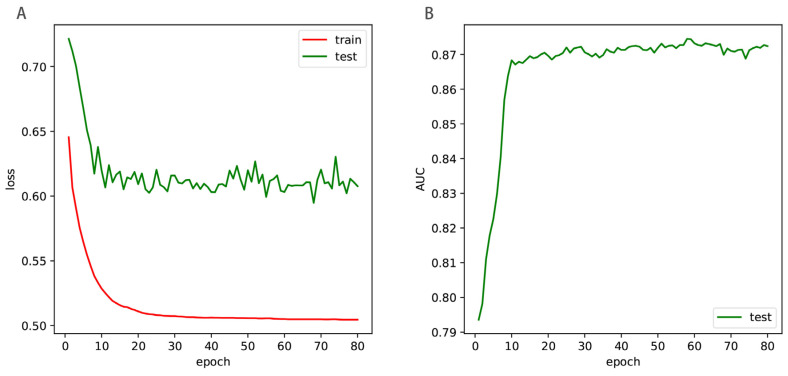
The loss curves (**A**) and the AUC curve of the independent test (**B**). The ‘train’ denoted the training loss in the benchmark dataset, and the ‘test’ denoted the loss in the training set and AUC values of the independent test.

In the Enhancer-LSTMAtt, there are up to 250,921 trainable parameters. The more trainable parameters there are, the more overfitting the deep learning model. We used dropout and batch normalization to reduce model overfitting. We investigated the roles of both techniques in reducing overfitting. As shown in Figure 7, the training loss descended rapidly at the beginning stage and then slowly declined to be stable with the increment of epoch, while the loss of the independent test declined rapidly at the beginning stage and then fluctuated in a certain range. The AUC of the independent test ascended rapidly at the beginning stage and then tended to stabilize with the increment of epochs. Therefore, there is no remarkable overfitting issues for Enhancer-LSTMAtt.

Enhancers-LSTMAtt is a deep learning-based and end-to-end method that does not require any feature design. This avoided artificial interference and sophisticated feature extraction or selection. The Enhancers-LSTMAtt is easier to implement than the feature-based methods from this viewpoint. Most feature-based methods performed well over the cross validation but performed badly over the independent test, indicating the weakly generalized ability. For example, the EnhancerP-2L [51] achieved an MCC of 0.8340 and an MCC of 0.8398 over the 5-fold and 10-fold cross validations, respectively, but reached only an MCC of 0.5907, which decreased by more than 0.24. piEnPred [61] substantially decreased the MCC from 0.7660 over the 5-fold cross validation to 0.6099 over the independent test. The Enhancers-LSTMAtt did not reduce the MCC over the independent test and instead increased the MCC by at least 0.07. Thus, the Enhancers-LSTMAtt is more generalized to the independent test than the feature-based methods. Most deep learning-based methods either utilize the CNN, LSTM, or their combination for enhancer recognition. For example, both the iEnhancer-ECNN [50] and the iEnhancer-CNN [52] exploited the CNN, the iEnhancer-EBLSTM [59] used Bi-LSTM, and the DeployEnhancer [48] sequentially combined the CNN and Bi-LSTM. The CNN and Bi-LSTM are two popular neural network architectures that have the ability to capture different information. The sequential combination between CNN and Bi-LSTM is disadvantageous to complete exploitation of these two different types of information. Stacking the CNN and Bi-LSTM in a parallel manner is able to exploit their respective representations. In addition, we also used the residual network and the attention mechanism to improve representation. This is two potential reasons why Enhancers-LSTMAtt is superior to other deep learning-based methods in the independent test, such as the iEnhancer-ECNN [50], the iEnhancer-CNN [52], the iEnhancer-EBLSTM [59], and the DeployEnhancer [48]. On the other hand, inclusion of the residual neural network as well as the feed-forward attention and stacking CNN and Bi-LSTM in a parallel manner added complexity to a certain extent, which in turn increased the computing cost.

## 6. Conclusions

Identifying enhancers is key to uncovering their roles in the regulation of transcription for target genes. We employed multiple deep learning techniques (i.e., Bi-LSTM, CNN, residual network and feed-forward attention) to construct Enhancer-LSTMAtt for enhancer recognition. The Enhancer-LSTMAtt is of the following superiorities over the state-of-the-art methods: (1) the Enhancer-LSTMAtt stacked the CNN and the LSTM in parallel, not in a series-connection manner, which allows stacking diverse representations; (2) the Enhancer-LSTMAtt utilized the residual neural network, which allows construction of deeper neural networks without loss of information; and (3) the Enhancer-LSTMAtt employed the attention mechanism, which allows focusing on key information. Comprehensive comparison with state-of-the-art methods suggested that Enhancers-LSTMAtt was not only a stable tool but also an effective and efficient tool for enhancer identification.

## Figures and Tables

**Table 1:** The shapes of outputs and the numbers of parameters in the Enhancer-LSTMAtt.

Layers	Shape of Output	Number of Parameters
Input	(None, 200)	0
Embedding	(None, 200, 32)	160
Conv1D(32, 8)	(None, 200, 32)	8224
Batch Normalization	(None, 200, 32)	128
Activation	(None, 200, 32)	0
Max Pooling	(None, 199, 32)	0
Conv1D_1(64, 8)	(None, 96, 64)	16,448
Batch Normalization	(None, 96, 64)	256
Activation	(None, 96, 64)	0
Conv1D_2(64, 8)	(None, 96, 64)	32,832
Batch Normalization	(None, 96, 64)	256
Conv1D_3(64, 8)	(None, 96, 64)	16,448
Add	(None, 96, 64)	0
Activation	(None, 96, 64)	0
Conv1D_4(64, 8)	(None, 96, 64)	32,832
Batch Normalization	(None, 96, 64)	256
Activation	(None, 96, 64)	0
Conv1D_5(64, 8)	(None, 96, 64)	32,832
Batch Normalization	(None, 96, 64)	256
Add	(None, 96, 64)	0
Activation	(None, 96, 64)	0
Conv1D_6(64, 8)	(None, 96, 64)	32,832
Batch Normalization	(None, 96, 64)	256
Activation	(None, 96, 64)	0
Conv1D_7(64, 8)	(None, 96, 64)	32,832
Batch Normalization	(None, 96, 64)	256
Add	(None, 96, 64)	0
Activation	(None, 96, 64)	0
Global Max Pooling	(None, 64)	0
Bidirectional LSTM	(None, 200, 64)	16,640
Bidirectional LSTM	(None, 200, 64)	24,832
Attention	(None, 64)	264
Dropout	(None, 64)	0
Concatenate	(None, 128)	0
Dense(16)	(None, 16)	2064
Dense(1)	(None, 1)	17

**Table 2:** Performances of 5-fold cross validation.

	SN	SP	ACC	MCC	AUC
Frist Stage					
fold 1	0.7374	0.7811	0.7593	0.5190	0.8281
fold 2	0.8013	0.7576	0.7795	0.5595	0.8388
fold 3	0.6801	0.8350	0.7576	0.5214	0.8114
fold 4	0.7710	0.7980	0.7845	0.5692	0.8454
fold 5	0.6622	0.8311	0.7466	0.5004	0.8056
mean	0.7304	0.8006	0.7655	0.5339	0.8259
Second Stage					
fold 1	0.6846	0.6510	0.6678	0.3358	0.6780
fold 2	0.6913	0.5369	0.6141	0.2310	0.6131
fold 3	0.7297	0.5811	0.6554	0.3143	0.6392
fold 4	0.6149	0.6419	0.6284	0.2569	0.6405
fold 5	0.6622	0.6014	0.6318	0.2640	0.6488
mean	0.6765	0.6024	0.6395	0.2804	0.6439

**Table 3:** Summary of the state-of-the-art methods for predicting enhancers.

Method	Jackknife	5-Fold	10-Fold	Independent	Enhancer or Not	Strong or Weak
iEnhancer-2L [40]	√			√	√	√
iEnhancer-PsedeKNC [41]		√			√	√
EnhancerPred [42]	√			√	√	√
EnhancerPred2.0 [43]	√				√	√
Enhancer-Tri-N [44]	√				√	√
iEnhaner-2L-Hybrid [45]	√				√	√
iEnhancer-EL [46]	√			√	√	√
iEnhancer-5Step [47]		√		√	√	√
DeployEnhancer [48]		√		√	√	√
ES-ARCNN [49]			√	√		√
iEnhancer-ECNN [50]				√	√	√
EnhancerP-2L [51]		√	√	√	√	√
iEnhancer-CNN [52]		√		√	√	√
iEnhancer-XG [53]			√	√	√	√
Enhancer-DRRNN [54]				√	√	√
Enhancer-BERT [55]		√		√	√	
iEnhancer-KL [56]		√			√	√
iEnhancer-RF [57]		√		√	√	√
spEnhancer [58]				√	√	√
iEnhancer-EBLSTM [59]				√	√	√
iEnhancer-GAN [60]			√	√	√	√
piEnPred [61]		√		√	√	√
iEnhancer-RD [62]		√		√	√	√
iEnhancer-MFGBDT [63]			√	√	√	√

√ denoted conduction of corresponding cross validation.

**Table 4:** Comparison with state-of-the-art methods by 5-fold cross validation.

	SN	SP	ACC	MCC	AUC
Frist Stage					
iEnhancer-PsedeKNC [41]	0.7731	0.7630	0.7678	0.5400	0.8500
iEnhancer-5Step [47]	0.8110	0.8350	0.8230	0.6500	-
DeployEnhancer [48]	0.7325	0.7642	0.7483	0.4980	0.7694
EnhancerP-2L [51]	0.9077	0.9259	0.9168	0.8340	0.9400
iEnhancer-CNN [52]	0.7588	0.8888	0.8063	0.6929	0.8957
Enhancer-BERT [55]	0.7950	0.7300	0.7620	0.5250	-
iEnhancer-KL [56]	0.8322	0.8524	0.8423	0.6800	-
iEnhancer-RF [57]	0.7364	0.7871	0.7618	0.5264	0.8400
piEnPred [61]	0.9228	0.8047	0.8788	0.7660	0.9603
iEnhancer-RD [62]	0.8100	0.7650	0.7880	0.5760	0.8440
Enhancer-LSTMAtt	0.7304	0.8006	0.7655	0.5339	0.8259
Second Stage					
iEnhancer-PsedeKNC [41]	0.6262	0.6441	0.6341	0.2700	0.6900
iEnhancer-5Step [47]	0.7530	0.6080	0.6810	0.3700	-
DeployEnhancer [48]	0.7965	0.3828	0.5896	0.1970	0.6068
EnhancerP-2L [51]	0.6221	0.6182	0.6193	0.2400	0.9000
iEnhancer-CNN [52]	0.7364	0.7680	0.7643	0.4505	0.8109
iEnhancer-KL [56]	0.9340	0.9287	0.9313	0.8600	-
iEnhancer-RF [57]	0.6846	0.5661	0.6253	0.2529	0.6700
piEnPred [61]	0.6554	0.7094	68.24	0.3654	0.7568
iEnhancer-RD [62]	0.8400	0.5700	0.7050	0.4260	0.7920
Enhancer-LSTMAtt	0.6765	0.6024	0.6395	0.2804	0.6439

**Table 5:** Comparison with state-of-the-art methods by 10-fold cross validation.

	SN	SP	ACC	MCC	AUC
Frist Stage					
EnhancerP-2L [51]	0.8653	0.9690	0.9172	0.8398	0.9700
iEnhancer-XG [53]	0.7570	0.8650	0.8110	0.6265	-
iEnhancer-GAN [60]	0.9510	0.9510	0.9510	0.9020	-
iEnhancer-MFGBDT [63]	0.7754	0.7978	0.7867	0.5735	-
Enhancer-LSTMAtt	0.7414	0.7873	0.7658	0.5298	0.8256
Second Stage					
ES-ARCNN [49]	0.7278	59.57	66.17	0.3263	0.6604
EnhancerP-2L [51]	0.8049	0.9397	0.8723	0.7519	0.9300
iEnhancer-XG [53]	0.7494	0.5855	0.6674	0.3395	-
iEnhancer-GAN [60]	0.8730	0.8710	0.8720	0.7440	-
iEnhancer-MFGBDT [63]	0.7056	0.6163	0.6604	0.3232	-
Enhancer-LSTMAtt	0.6463	0.6380	0.6429	0.2851	0.6550

**Table 6:** Comparison with state-of-the-art methods by independent test.

	SN	SP	ACC	MCC	AUC
Frist Stage					
iEnhancer-2L [40]	0.7100	0.7500	0.7300	0.4604	0.8062
EnhancerPred [42]	0.7350	0.7450	0.7400	0.4800	0.8013
iEnhancer-EL [46]	0.7100	0.7850	0.7475	0.4964	0.8173
iEnhancer-5Step [47]	0.8200	0.7600	0.7900	0.5800	-
DeployEnhancer [48]	0.7550	0.7600	0.7550	0.5100	0.7704
iEnhancer-ECNN [50]	0.7520	0.7850	0.7690	0.5370	0.8320
EnhancerP-2L [51]	0.7810	0.8105	0.7950	0.5907	-
iEnhancer-CNN [52]	0.7825	0.7900	0.7750	0.5850	-
iEnhancer-XG [53]	0.7400	0.7750	0.7575	0.5150	-
Enhancer-DRRNN [54]	0.7330	0.8010	0.7670	0.5350	0.8370
Enhancer-BERT [55]	0.8000	0.7120	0.7560	0.5140	-
iEnhancer-RF [57]	0.7850	0.8100	0.7975	0.5952	0.8600
spEnhancer [58]	0.8300	0.7150	0.7725	0.5793	0.8235
iEnhancer-EBLSTM [59]	0.7550	0.7950	0.7720	0.5340	0.8350
iEnhancer-GAN [60]	0.8110	0.7580	0.7840	0.5670	-
piEnPred [61]	0.8250	0.7840	0.8040	0.6099	-
iEnhancer-RD [62]	0.8100	0.7650	0.7880	0.5760	0.8440
iEnhancer-MFGBDT [63]	0.7679	0.7955	0.7750	0.5607	-
Enhancer-LSTMAtt	0.7950	0.8150	0.8050	0.6101	0.8588
Second Stage					
iEnhancer-2L [40]	0.4700	0.7400	0.6050	0.2181	0.6678
EnhancerPred [42]	0.4500	0.6500	0.5500	0.1020	0.5790
iEnhancer-EL [46]	0.5400	0.6800	0.6100	0.2222	0.6801
iEnhancer-5Step [47]	0.7400	0.5300	0.6350	0.2800	-
DeployEnhancer [48]	0.8315	0.4561	0.6849	0.3120	0.6714
ES-ARCNN [49]	0.8600	0.4500	0.6560	0.3399	-
iEnhancer-ECNN [50]	0.7910	0.5640	0.6780	0.3680	0.7480
EnhancerP-2L [51]	0.6829	0.7922	0.7250	0.4624	-
iEnhancer-CNN [52]	0.6525	0.7610	0.7500	0.3232	-
iEnhancer-XG [53]	0.7000	0.5700	0.6350	0.2720	-
Enhancer-DRRNN [54]	0.8580	0.8400	0.8490	0.6990	-
iEnhancer-RF [57]	0.9300	0.7700	0.8500	0.7091	0.9700
spEnhancer [58]	0.9100	0.3300	0.6200	0.3703	0.6253
iEnhancer-EBLSTM [59]	0.8120	0.5360	0.6580	0.3240	0.6880
iEnhancer-GAN [60]	0.9610	0.5370	0.7490	0.5050	-
piEnPred [61]	0.7000	0.7500	0.7250	0.4506	-
iEnhancer-RD [62]	0.8400	0.5700	0.7050	0.4260	0.7920
iEnhancer-MFGBDT [63]	0.7255	0.6681	0.6850	0.3862	-
Enhancer-LSTMAtt	0.9900	0.8000	0.8950	0.8047	0.9637

## Data Availability

https://github.com/feng-123/Enhancer-LSTMAtt.
